# Do women accurately predict their odds of having a child following planned oocyte cryopreservation?

**DOI:** 10.1530/RAF-24-0118

**Published:** 2025-02-27

**Authors:** Matan Friedman, Nadine Jaffe, Daniel Tairy, Maya Torem, Arieh Raziel, Maya Finkelstein, Eran Horowitz, Ariel Weissman, Yossi Mizrachi

**Affiliations:** ^1^ Fertility Unit, The Edith Wolfson Medical Center, Holon, Israel; ^2^ Faculty of Medical and Health Sciences, Tel-Aviv University, Tel-Aviv, Israel

**Keywords:** ART, planned oocyte cryopreservation, fertility preservation, survey

## Abstract

**Graphical abstract:**

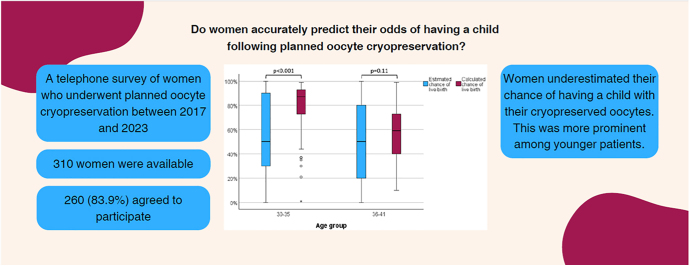

**Abstract:**

Planned oocyte cryopreservation (POC) has become widely available, allowing women to circumvent age-related fertility decline. The aim of our study was to examine whether women who have undergone POC were able to correctly predict the chance of having a child with their cryopreserved oocytes. We conducted a telephone survey with 260 women who underwent POC at our center between January 2017 and December 2023. Participants were asked to estimate their chance of having at least one live birth in case they would use their cryopreserved oocytes. For each participant, we also calculated the chance of achieving at least one live birth based on her age at the last oocyte retrieval and the number of cryopreserved oocytes, according to a model published by Goldman and colleagues in 2017. The median estimated probability of achieving a live birth was 50%, while the median calculated probability was 75% (*P* < 0.001). Only 28.1% of the participants accurately estimated their chances. In conclusion, a large percentage of women undergoing POC underestimate the probability of achieving a live birth if they use their cryopreserved oocytes. Improved counseling is essential to provide comprehensive information about the probability of live birth and prevent women from undergoing unnecessary treatments.

**Lay summary:**

More and more women choose to freeze eggs in order to circumvent age-related fertility decline. In this study, we asked women to estimate their chance of having a child if they used their frozen eggs in the future. For each patient, the chance was also calculated based on a model taking into account the number of eggs she had frozen and her age during egg freezing. Surprisingly, women estimated their chance of having a child as being lower than it actually is. Our results highlight the importance of providing patients with clear information about treatment success to prevent them from undergoing unnecessary additional treatments.

## Introduction

Planned oocyte cryopreservation (POC) has become widely available, allowing women to preserve their fertility in an attempt to circumvent age-related fertility decline (ASRM 2018, Johnston *et al.* 2021). The main reasons for women to pursue POC are increasing age and the absence of a male partner committed to start a family (Inhorn *et al.* 2018, Baldwin *et al.* 2019*a*, Seyhan *et al.* 2021, Yee *et al.* 2021). When patients consider how many cycles of POC they should do, they need to receive clear counseling, including an accurate estimation of the chance to achieve a live birth. Overestimating the chance of a live birth might result in a sense of ‘false hope’ and the cryopreservation of an insufficient number of oocytes. On the other hand, underestimating the chance of a live birth might result in a sense of urgency and unjustified financial, physical and psychological burdens (Devine *et al.* 2015, ASRM 2018, Baldwin & Culley 2020).

Previous studies have consistently shown that the chance of having a child with cryopreserved oocytes depends on two major factors: age at oocyte retrieval and the number of cryopreserved oocytes (Cobo *et al.* 2015, 2018, Cascante *et al.* 2022, Hirsch *et al.* 2024). In 2017, Goldman *et al.* (2017) published an evidence-based model to predict the probability of a woman to achieve a live birth based on her age at oocyte retrieval and the number of cryopreserved oocytes. That model was derived from a surrogate population of ICSI patients with uncompromised ovarian reserve.

Previous surveys on women undergoing POC have focused on their clinical characteristics, motivations and satisfaction levels (Baldwin *et al.* 2015, 2019*b*, Greenwood *et al.* 2018, Baldwin & Culley 2020, Jones *et al.* 2020, Wafi *et al.* 2020, Seyhan *et al.* 2021). However, little is known about how accurately they perceive their odds of having a child with their cryopreserved oocytes. In the current study, we conducted a telephone survey among women who underwent POC at a single fertility unit, focusing on their self-estimation of the odds to achieve at least one live birth by utilizing their cryopreserved oocytes in the future. The aim of our study was to examine whether women who have undergone POC were able to correctly predict the chance of having a child with their cryopreserved oocytes and to identify factors that are associated with over- and underestimation.

## Materials and methods

In this cross-sectional study, we conducted a telephone survey among women who underwent one or more cycles of POC between January 2017 and December 2023 at a single university-affiliated fertility unit. The survey was conducted between February 2024 and May 2024. Women were eligible for participation if they underwent planned (elective, nonmedical) oocyte cryopreservation for age-related fertility decline. Those who had undergone medically indicated fertility preservation (e.g., before gonadotoxic treatment) and those who had undergone embryo cryopreservation were excluded. The study was approved by the Institutional Review Board (IRB, approval number 0118-23-WOMC, November 26th, 2023). Women gave verbal consent for participation, which was documented as required by the IRB for telephone surveys.

POC was approved in Israel in 2011 for women aged 30–41, with a limit of up to four ovarian stimulation cycles, which was later expanded to six cycles. Most POC treatments in Israel are not covered by national health insurance and are self-funded. Only a small percentage of women with low ovarian reserve, according to baseline follicle stimulating hormone (FSH) level, anti-Müllerian hormone (AMH) level and antral follicle count, are entitled for reimbursement.

A 13-item questionnaire was created by the research group. The content of the questionnaire items was derived from the up-to-date literature on POC, including questions addressing demographics, satisfaction, perceptions and estimation of the chances of usability and success. The two main questions were: ‘how do you estimate the chances that you will use your cryopreserved oocytes in the future?’ and ‘if you do decide to use your eggs, what do you think are your chances of having at least one successful childbirth?’. The survey was conducted by five fertility specialists after training in order to achieve consistency and uniformity in the way the questions were presented.

The primary outcome of the study was the ‘estimated’ chance of having at least one live birth, as predicted by the participants, in case they would use their cryopreserved oocytes. For each participant, the ‘calculated’ chance of achieving at least one live birth was also determined based on her age at the last oocyte retrieval and the number of cryopreserved oocytes, according to Goldman *et al.* (2017). The estimated chance was considered accurate if the difference between the estimated and calculated chances was no greater than ±10%. Overestimation was defined when the estimated chance was >10% higher than the calculated chance. Underestimation was defined when the estimated chance was >10% lower than the calculated chance. Secondary outcomes included personal satisfaction and future reproductive plans.

Statistical analysis was performed using SPSS version 28.0 (IBM Corp., USA; https://www.ibm.com/products/spss-statistics). Continuous variables are presented as mean ± standard deviation (SD) of median (range), as appropriate. Categorical variables are presented as numbers and percentages. The Wilcoxon test for paired nonparametric variables was used for the comparison of the estimated and calculated chances of a live birth. The characteristics of women who overestimated their chances of having a child with their cryopreserved oocytes were compared with those who underestimated their chances. Comparison of non-paired continuous variables was performed by Student’s *t*-test and comparison of categorical variables was performed by the chi-squared test or Fischer’s exact test, as appropriate.

We anticipated that women would estimate the chance of having a live birth as around 50%. Based on that, a sample size of 267 participants would be sufficient to detect a 12% difference between the estimated and calculated chance of live birth, with a power of 80% and an alpha of 0.05. A *P*-value <0.05 was considered statistically significant.

## Results

During the study period (2017–2023), 435 women underwent POC at our unit. We managed to contact 310 women via phone call, out of whom 260 (83.9%) consented to participate in the study. The median follow-up time was 15 months (range: 4–84). The mean age of the participants at the time of their last ovum pick-up (OPU) was 35.3 ± 2.3 years (range: 30–41).

Baseline characteristics are presented in Table 1. About half of the participants (51.2%) underwent a single oocyte retrieval. The mean total number of cryopreserved MII oocytes was 17.6 ± 9.7 (range: 2–67). The treatment was self-funded in most (93.5%) patients. In terms of education level, most patients had an academic degree while undergoing POC. The main reason for choosing to freeze oocytes was the absence of a partner to start a family with (88.5%).

**Table 1:** Baseline characteristics of women who underwent POC. Data are presented as mean ± SD or *n* (%).

Characteristics	Values
Age at last oocyte retrieval (years)	35.3 ± 2.3
Age at the time of survey (years)	37.0 ± 2.7
Number of stimulation cycles	
1	133 (51.2%)
2	92 (35.4%)
3	24 (9.2%)
4	11 (4.2%)
Total number of cryopreserved oocytes	17.6 ± 9.7
Self-funded treatment	243 (93.5%)
Number of women having no children at the time of POC	256 (98.5%)
Highest level of education at the time of POC	
Primary school	1 (0.4%)
High school	37 (14.2%)
Bachelor’s degree	125 (48.1%)
Master’s degree or higher	97 (37.3%)
Marital status at the time of POC	
Single	238 (91.5%)
In a relationship	12 (4.6%)
Divorced/separated	10 (3.8%)
Main reason for undergoing POC	
I did not have a partner committed to start a family	230 (88.5%)
I was not ready to have children	19 (7.3%)
I was keen to have a big family	10 (3.8%)
I wanted to focus on my career	1 (0.4%)

POC, planned oocyte cryopreservation; SD, standard deviation.

At the time of the survey, three patients (1.2%) had already used their cryopreserved oocytes in order to conceive, seven patients (2.7%) had conceived with a new ART treatment and 28 patients (10.8%) had conceived naturally.

Table 2 presents the responses to questions related to satisfaction and future reproductive plans. Most participants (85.4%) announced that they were satisfied with their decision to freeze oocytes, and 96.5% said they would recommend POC to a friend. More than half (55.4%) of the respondents said they would rather perform the POC at an earlier age. Overall, 170 (65.4%) participants estimated their chances of using their cryopreserved oocytes in the future as moderate or high. In the advanced age group (>35 yeas), more women estimated the chance of future use to be moderate or high compared to the younger age group (72.6 vs 60.4%, *P* = 0.041). Most women (61.2%) said they would consider sperm donation in case they do not have a partner.

**Table 2:** Satisfaction and future reproductive plans among women who underwent POC. Data are presented as *n* (%).

Questions	Answers
Are you satisfied regarding your decision to freeze oocytes or regret it?	
Satisfied	222 (85.4%)
Regret	6 (2.3%)
Do not know	32 (12.3%)
Would you recommend a friend to freeze oocytes?	
Yes	251 (96.5%)
No	9 (3.5%)
What is your opinion about the timing of POC	
The timing was good	114 (43.8%)
I would rather do it at an earlier age	144 (55.4%)
I would rather not do it at all	(0.8%)
To what extent did the treatment involve difficulties?	
No difficulty	52 (20.0%)
Mild difficulty	89 (34.2%)
Moderate difficulty	68 (26.2%)
Severe difficulty	51 (19.6%)
How do you estimate the chances that you will use your cryopreserved oocytes in the future?	
I have already used my cryopreserved oocytes	1 (0.4%)
Low	18 (6.9%)
Fair	61 (23.5%)
Moderate	116 (44.6%)
High	54 (20.8%)
Do not know	10 (3.8%)
Ideally, how many children would you like to have?	
1	7 (2.7%)
2	133 (51.2%)
3	69 (26.5%)
>3	35 (13.5%)
Do not know	16 (6.2%)
In case you do not have a partner, would you consider using a donor sperm?	
Yes	159 (61.2%)
No	44 (16.9%)
Do not know	57 (21.9%)

POC, planned oocyte cryopreservation.

**Figure 1:**
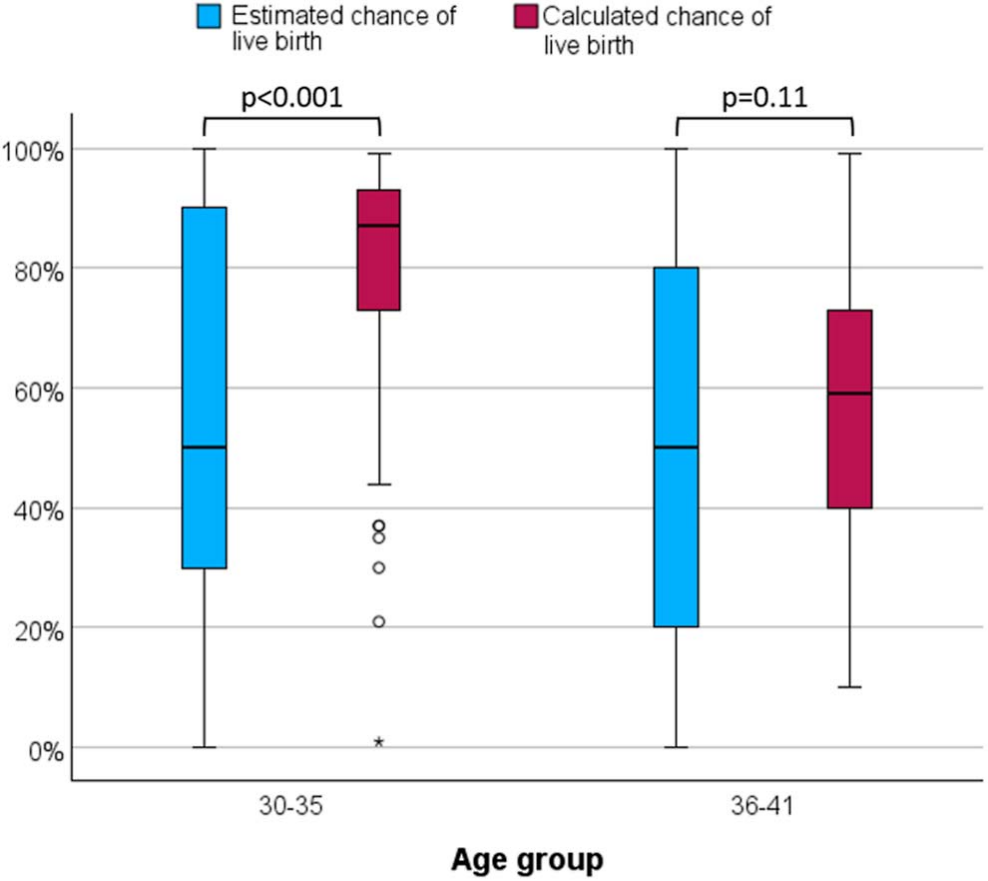
Comparison of the estimated and calculated chances of a live birth stratified by age group. A box plot presenting the estimated and calculated chances of a live birth in women who underwent POC. The solid lines indicate the medians, and the boxes indicate the interquartile range. Younger patients underestimated their chance of a live birth, whereas older patients had a more accurate estimation of their actual chance.

Using the Wilcoxon test for paired nonparametric variables, we found that patients underestimated their chance of having a child with their cryopreserved oocytes. Their median estimated chance was 50%, whereas the median calculated chance was 75.0% (*P* < 0.001). This trend remained significant among women who underwent POC at the age of 30–35. In contrast, in women who underwent POC at age >35, the difference between the estimated and the calculated chances did not reach statistical significance (Fig. 1).

Only 73 respondents (28.1%) accurately predicted their chance of having a child with their cryopreserved oocytes, whereas 138 (53.1%) respondents underestimated their chances and 44 (16.9%) overestimated their chances. Five respondents replied that they did not know what their chances were. Compared to women who overestimated their chances, women who underestimated their chances were younger at the time of their last oocyte retrieval and at the time of the survey. They also had a higher number of cryopreserved oocytes (Table 3).

**Table 3:** Comparison of women who over- and under- estimated their chances of achieving a live birth with their cryopreserved oocytes. Data are presented as mean ± SD or *n* (%).

	Overestimation (*n* = 44)	Underestimation (*n* = 138)	*P*-value
Age at last POC cycle (years)	37.1 ± 2.5	34.7 ± 2.0	<0.001
Age at time of survey (years)	39.2 ± 2.4	36.3 ± 2.4	<0.001
More than one stimulation cycle	22 (50.0%)	62 (44.9%)	0.55
Total number of cryopreserved oocytes	13.4 ± 7.0	16.8 ± 9.1	0.01
Tertiary education or above	34 (77.3%)	122 (88.4%)	0.066
Experienced any adversities during the process (physical, emotional or financial)	32 (72.7%)	112 (81.2%)	0.23
Ideal number of desired children >2	16 (36.4%)	54 (39.1%)	0.74
Willing to use sperm donation	26 (59.1%)	89 (64.5%)	0.51

SD, standard deviation.

## Discussion

In this study, we found that only 28.1% of women who underwent POC accurately predicted their chances of having a child using their cryopreserved oocytes in the future. The majority of women underestimated their chances, with underestimation being more pronounced among those who underwent POC at a younger age and had a high number of oocytes cryopreserved.

In the last decades, women have been having children at older ages. This trend is driven by greater gender equality and expanded opportunities for women and is further facilitated by the availability of contraception and assisted reproductive technologies (Lemoine & Ravitsky 2015). Along with improvements in oocyte cryopreservation techniques, POC has become widely available, enabling individuals to extend their reproductive window. A major increase in the number of POC cycles was reported in the USA, Australia and New Zealand (Johnston *et al.* 2021), as well as in Europe (Martinez 2017).

While the technology is promising, POC may be involved with substantial financial, psychological and physical difficulties (Baldwin & Culley 2020). Therefore, providers must ensure that women requesting POC are informed about its efficacy, safety, costs, benefits and risks. It is important that clinicians provide counseling about the probability that an individual patient will attempt to use her cryopreserved oocytes and the chance of achieving a live birth if she does. A realistic estimation will allow each patient to decide on her target number of cryopreserved oocytes and, accordingly, the number of POC cycles she should opt for (ASRM 2018).

In our study, the majority of respondents did not estimate their chance of having a child correctly. It appears that women did not comprehend the large impact that age has on success rates. Numerous studies have demonstrated that a patient’s age at cryopreservation greatly affects the chance of achieving a live birth. A fifteen-years follow-up of 543 patients who attempted to use their cryopreserved oocytes found that the final live birth rate (FLBR) was 51% in women who were <38 years old at cryopreservation. In contrast, the FLBR was 34% in women aged 38–40 and only 23% in women aged ≥41 at oocyte cryopreservation (Cascante *et al.* 2022). Similarly, a recent systematic review and meta-analysis including 1,517 women who attempted to conceive with their cryopreserved oocytes (Hirsch *et al.* 2024) found that the live birth rate per patient was 52% in women aged ≤35, 34% in women aged 36–39 and only 19% in women aged >40 at the time of oocyte cryopreservation. These data emphasize the importance of a patient’s age at cryopreservation and should be discussed with women considering how many POC cycles they should undergo.

Evidence regarding women’s estimation of the chances to have a child with their cryopreserved oocytes is limited. Seyhan *et al.* (2021) conducted a survey among 133 women who underwent POC. Participants were asked, ‘what is the pregnancy chance per one frozen egg in women between 38 and 40 years?’. Similar to our study, only 28% of patients estimated the chance correctly. In a cohort of 85 women who underwent POC in the UK (Jones *et al.* 2020), the majority of women (83%) knew there was a chance of treatment failure in the future and that a live birth could not be guaranteed. To the best of our knowledge, in previous studies, women were not asked to quantify their own chances.

Previous studies have shown that only a small portion of women return to use their cryopreserved oocytes. A retrospective review from over a decade of POC at a single large center in the US found that only 7.4% of patients returned to use their cryopreserved oocytes (Leung *et al.* 2021). Similarly, a systematic review including ten studies conducted between 1999 and 2020 found a return rate of 11.1 ± 4.7% (Hirsch *et al.* 2024). While these rates might increase with longer follow-up, they still indicate that most patients will not return to utilize their oocytes. The most common reasons for not using the cryopreserved oocytes are achieving spontaneous pregnancy or preferring not to have a child without a partner (Tsafrir *et al.* 2021). In our study, 65.4% of the participants estimated the chances of using their cryopreserved oocytes in the future as moderate or high. A previous survey from Belgium also reported that two-thirds of women who had POC anticipated using their oocytes at some point in the future (Wafi *et al.* 2020). This is, of course in contrast, with the above reports of the return rate, reflecting a misunderstanding of the actual chance of natural conception.

The misconceptions that they will likely return to use their cryopreserved oocytes and that the chances of having a child with these oocytes are low might prompt women to undergo additional unnecessary treatments, thereby increasing their financial, physical and psychological burdens (Baldwin & Culley 2020). Indeed, most respondents said that the treatment involved some difficulties. On the other hand, a small number of older patients (16.9%) overestimated their chances of a live birth. This might generate a sense of ‘false hope’, resulting in the cryopreservation of an insufficient number of oocytes and delaying pregnancy to a point where women might not be able to conceive with their own oocytes. Both types of misconceptions should be avoided by providing clear and comprehensive counseling.

Our study demonstrated high levels of satisfaction among women who chose to undergo POC. Moreover, the vast majority of participants indicated they would recommend POC to a friend. These results suggest that women who opt for oocyte cryopreservation understand the biological rationale behind the procedure and are highly satisfied with their decision to invest time and money in extending their fertility window. Our results are in agreement with previous surveys also showing high levels of satisfaction (Greenwood *et al.* 2018, Jones *et al.* 2020, Seyhan *et al.* 2021, Jaswa *et al.* 2023).

The strengths of our study include a large cohort of patients and a high response rate. Furthermore, we addressed the question of whether women accurately estimate their chances of success if they will use their cryopreserved oocytes in the future, a topic that has been scarcely examined in previous research. Nevertheless, some limitations should be acknowledged. The main limitation of our study arises from the fact that the calculation of the probability of a live birth was based on the model of Goldman *et al.* which is purely theoretical and was derived from a specific IVF program with patients undergoing treatment for infertility. We, like Goldman *et al.*, suggest that the best outcome data to develop a predictive model should be obtained from women who have undergone POC and then returned to use their oocytes. However, at the present time, there is a paucity of such validation data available. In addition, we approached women who underwent POC at a single fertility unit, which might affect the generalizability of our results. However, the baseline characteristics of the participants in our cohort are similar to those of previous cohorts of women undergoing POC. In addition, physicians’ counseling and other resources of information may differ between different clinics and countries.

In conclusion, women who undergo POC exhibit very high satisfaction rates. However, many underestimated their probability of achieving a live birth using their cryopreserved oocytes. Improved counseling is essential to provide comprehensive information and prevent women from undergoing unnecessary treatments.

## Declaration of interest

The authors declare that there is no conflict of interest that could be perceived as prejudicing the impartiality of the work reported.

## Funding

This research did not receive any specific grant from any funding agency in the public, commercial or not-for-profit sector.

## Author contribution statement

M Friedman contributed to the writing of the manuscript, as well as to data collection and analysis. N Jaffe assisted with study design, data collection and curation. D Tairy and M Torem assisted with data collection and analysis. M Finkelstein, E Horowitz, A Weissman and A Raziel helped design the study, write and review the manuscript while providing valuable input for improvement. Y Mizrachi contributed to designing the study, writing the manuscript, collecting data and conducting the statistical analysis.
